# Roadmap towards zero leprosy, Pakistan

**DOI:** 10.2471/BLT.24.292585

**Published:** 2025-06-10

**Authors:** Anil Fastenau, Ali Murtaza, Abdul Salam, Muhammed Iqbal, Nimer Ortuño-Gutiérrez, Fabian Schlumberger, Sophie CW Unterkircher, Elias Treml, Thomas Hambridge, Epco Hasker, Chris Schmotzer, Paul Saunderson

**Affiliations:** a German Leprosy and Tuberculosis Relief Association, Raiffeisenstrasse 3, 97080 Wuerzburg, Germany.; b Marie Adelaide Leprosy Center, Karachi, Pakistan.; c Damien Foundation, Brussels, Belgium.; d Institute of Public Health and Nursing Research, University of Bremen, Bremen, Germany.; e Erasmus MC, University Medical Centre Rotterdam, Rotterdam, Kingdom of the Netherlands.; f Department of Public Health, Institute of Tropical Medicine, Antwerp, Belgium.; g Rawalpindi Leprosy Hospital, Rawalpindi, Pakistan.; h American Leprosy Missions, Greenville, United States of America.

## Abstract

The World Health Organization recently redefined leprosy elimination as a phased process, with the first milestone being the interruption of transmission, achieved when no new child cases (defined as younger than 15 years) are reported for five consecutive years. In Pakistan, the well-functioning leprosy programme, with effective case management, context-specific active case-finding strategies and a robust data management system, has contributed to a decrease in new cases. Between 2001 and 2023, new adult cases dropped by 75% (from 878 cases to 220 cases annually) and child cases by 83% (from 93 to 16). To support the country’s goal of no new child cases by 2030 and ultimately eliminate the disease, the nongovernmental organizations Marie Adelaide Leprosy Centre and Aid to Leprosy Patients, with support from the German Leprosy and Tuberculosis Relief Association, have developed a zero leprosy roadmap. As part of this roadmap, the leprosy elimination strategy emphasizes improving active case-finding and providing post-exposure prophylaxis for contacts of leprosy cases, who are at the highest risk. Other key activities include establishing a monitoring and evaluation system for leprosy elimination, upgrading the health information management system to DHIS2, and training general practitioners and dermatologists to improve their capacity for accurate diagnosis and referral. The strategy also emphasizes improved counselling for new cases and the active involvement of individuals affected by leprosy in policy discussions. The roadmap offers globally relevant, scalable strategies for leprosy elimination in low-endemic settings. Lessons from Pakistan’s experience can inform and inspire similar efforts in other countries.

## Résumé

L’Organisation mondiale de la Santé a récemment redéfini l’élimination de la lèpre comme un processus progressif, dont la première étape est l’interruption de la transmission, obtenue lorsqu’aucun nouveau cas chez un enfant (défini comme âgé de moins de 15 ans) n’est signalé pendant cinq années consécutives. Au Pakistan, le bon fonctionnement du programme de lutte contre la lèpre, qui s’articule autour d’une gestion efficace des cas, autour de stratégies actives de recherche de cas adaptées au contexte et autour d’un système solide de gestion des données, a contribué à la diminution du nombre de nouveaux cas. De 2001 à 2023, les nouveaux cas chez les adultes ont chuté de 75% (de 878 à 220 cas par an) et les cas chez les enfants de 83% (de 93 à 16). Pour soutenir l’objectif du pays de ne plus enregistrer de nouveaux cas chez les enfants d’ici à 2030 et, à terme, d’éradiquer la maladie, les organisations non gouvernementales Marie Adelaide Leprosy Centre et Aid to Leprosy Patients, avec le soutien de l’association allemande de lutte contre la lèpre et la tuberculose, ont élaboré une feuille de route d’éradication de la lèpre. Dans le cadre de cette feuille de route, la stratégie d’éradication de la lèpre met l’accent sur l’amélioration de la recherche active de cas et sur la fourniture d’une prophylaxie post-exposition aux contacts des cas de lèpre, qui présentent le risque le plus élevé. D’autres activités clés comprennent la mise en place d’un système de suivi et d’évaluation pour l’éradication de la lèpre, la mise à niveau du système de gestion des informations sanitaires vers le DHIS2 et la formation des médecins généralistes et des dermatologues afin d’améliorer leur capacité à établir un diagnostic précis et à réorienter les patients. Cette stratégie met également l’accent sur l’amélioration des conseils pour les nouveaux cas et sur la participation active des personnes touchées par la lèpre aux débats politiques. La feuille de route propose des stratégies pertinentes et évolutives à l’échelle mondiale pour l’éradication de la lèpre dans les régions faiblement endémiques. Les leçons tirées de l’expérience pakistanaise permettent d’inspirer des efforts similaires dans d’autres pays.

## Resumen

La Organización Mundial de la Salud redefinió recientemente la eliminación de la lepra como un proceso por fases, cuyo primer hito es la interrupción de la transmisión, alcanzado cuando no se notifican nuevos casos en niños (definidos como menores de 15 años) durante cinco años consecutivos. En Pakistán, el programa de lepra, que funciona eficazmente gracias a una adecuada gestión de casos, estrategias de búsqueda activa adaptadas al contexto y un sólido sistema de gestión de datos, ha contribuido a reducir el número de nuevos casos. Entre 2001 y 2023, los nuevos casos en adultos disminuyeron un 75% (de 878 a 220 casos anuales) y los casos en niños un 83% (de 93 a 16). Para apoyar el objetivo nacional de no registrar nuevos casos en niños para 2030 y, en última instancia, eliminar la enfermedad, las organizaciones no gubernamentales Marie Adelaide Leprosy Centre y Aid to Leprosy Patients, con el apoyo de la German Leprosy and Tuberculosis Relief Association, han desarrollado una hoja de ruta hacia la eliminación total de la lepra. Como parte de esta hoja de ruta, la estrategia de eliminación pone énfasis en mejorar la búsqueda activa de casos y en la provisión de profilaxis posexposición a los contactos de personas con lepra, quienes presentan el mayor riesgo. Otras actividades clave incluyen el establecimiento de un sistema de monitoreo y evaluación para la eliminación de la lepra, la actualización del sistema de información en salud al DHIS2 y la capacitación de médicos generales y dermatólogos para mejorar su capacidad de diagnóstico preciso y remisión oportuna. La estrategia también subraya la importancia del acompañamiento psicosocial a nuevos casos y la participación activa de las personas afectadas por la lepra en las discusiones sobre políticas. Esta hoja de ruta ofrece estrategias escalables y de relevancia mundial para la eliminación de la lepra en contextos de baja endemicidad. Las lecciones aprendidas de la experiencia de Pakistán pueden orientar e inspirar esfuerzos similares en otros países.

## ملخص

أعادت منظمة الصحة العالمية مؤخرًا تعريف عملية القضاء على الجذام كعملية تدريجية، حيث تتمثل أولى خطواتها في وقف انتقال العدوى، ويتحقق ذلك عن طريق عدم اكتشاف أي حالات إصابة جديدة لدى الأطفال (الذين تقل أعمارهم عن 15 عامًا) لمدة خمس سنوات متتالية. في باكستان، ساهم البرنامج الفعال لمكافحة الجذام، بما يتضمنه من إدارة فعّالة للحالات، واستراتيجيات نشطة لاكتشاف الحالات حسب السياق، ونظام قوي لإدارة البيانات، في انخفاض عدد الحالات الجديدة. بين عامي 2001 و2023، انخفضت حالات الإصابة الجديدة بين البالغين بنسبة %75 (من 878 حالة إلى 220 حالة سنويًا)، وانخفضت حالات الإصابة بين الأطفال بنسبة %83 (من 93 حالة إلى 16 حالة). لدعم هدف الدولة المتمثل في القضاء التام على الجذام بين الأطفال بحلول عام 2030، والقضاء عليه في نهاية المطاف، وضعت المنظمتان غير الحكوميتين Marie Adelaide Leprosy Centre وAid to Leprosy Patients، بدعم من الجمعية الألمانية لإغاثة الجذام والسل، خارطة طريق للقضاء التام على الجذام. كجزء من هذه الخارطة، تُركز استراتيجية القضاء على الجذام على تحسين الكشف النشط عن الحالات وتوفير العلاج الوقائي بعد التعرض للمخالطين لحالات الجذام، الأكثر عرضة للخطر. وتشمل الأنشطة الرئيسية الأخرى إنشاء نظام رصد وتقييم للقضاء على الجذام، وتحديث نظام إدارة المعلومات الصحية إلى نظام DHIS2، وتدريب الأطباء العامين وأطباء الجلد لتحسين قدرتهم على التشخيص الدقيق والإحالة. كما تُركز الاستراتيجية على تحسين خدمات المشورة للحالات الجديدة، والمشاركة الفعالة للمصابين بالجذام في مناقشات السياسات. تُقدم خارطة الطريق استراتيجيات عالمية قابلة للتطوير للقضاء على الجذام في البيئات منخفضة التوطن. ويمكن للدروس المستفادة من تجربة باكستان أن تُثري وتُلهم جهودًا مماثلة في دول أخرى.

## 摘要

世界卫生组织最近重新定义了麻风病消除标准,将其分为多个阶段实施,其中第一个里程碑就是阻断传播,当连续五年内未报告任何新增儿童(年龄在 15 岁以下)病例时,即被视为实现了这一目标。在巴基斯坦,政府通过完善的麻风病防治计划以及有效的病例管理、因地制宜的主动病例发现策略和强大的数据管理系统减少了新增病例数量。在  2001 年至 2023 年期间,新增成人病例减少了 75%(即从每年 878 例减少至 220 例),新增儿童病例减少了 83%(即从每年 93 例减少至 16 例)。为了帮助巴基斯坦实现截至 2030 年无任何新增儿童病例的目标并最终消除麻风病,玛丽·阿德莱德麻风病中心 (Marie Adelaide Leprosy Centre) 和麻风病患者救助机构 (Aid to Leprosy Patients) 这两个非政府组织在德国麻风病与结核病救济协会 (German Leprosy and Tuberculosis Relief Association) 的支持下制定了实现零麻风病的路线图。作为该路线图的一部分,麻风病消除策略强调改进主动病例发现策略并为麻风病病例接触者(感染风险最高的人群)提供接触后预防治疗。其他主要活动包括构建麻风病消除监测和评估系统、将卫生信息管理系统升级至 DHIS2 以及针对全科医生和皮肤科医生开展相关培训以提升其准确诊断和转诊能力。该消除策略还强调提升新增病例咨询服务和主动邀请受麻风病影响的人群参与政策讨论。该路线图提供了适用于低流行地区麻风病消除工作且具有全球普适性和可扩展性的策略。从巴基斯坦麻风病消除工作中总结的经验教训可为其他国家的类似疾病消除工作提供相关信息和启示。

## Резюме

Всемирная организация здравоохранения недавно дала новое определение ликвидации лепры как поэтапного процесса, где первым этапом является прерывание передачи инфекции, определяемое как отсутствие новых случаев заболевания у детей (то есть у лиц моложе 15 лет) в течение непрерывного пятилетнего отчетного периода. В Пакистане хорошо функционирует программа борьбы с лепрой с эффективным ведением пациентов, с контекстно ориентированной стратегией выявления активных случаев и с надежной системой управления данными, что внесло свой вклад в снижение количества новых случаев. За период с 2001 по 2023 год число новых случаев заболевания сократилось у взрослых на 75% (с 878 до 220 случаев в год), а у детей на 83% (с 93 до 16). Чтобы поддержать страну в достижении ее цели, то есть отсутствия новых случаев заболевания у детей к 2030 году, неправительственные организации «Центр по борьбе с лепрой им. Марии Аделаиды» (Marie Adelaide Leprosy Centre) и «Помощь пациентам с лепрой» (Aid to Leprosy Patients) при поддержке Немецкой ассоциации помощи больным лепрой и туберкулезом (German Leprosy and Tuberculosis Relief Association) разработали план действий по ликвидации лепры. В рамках этого плана стратегия элиминации лепры подчеркивает важность усиленного выявления активных случаев и предоставления профилактического лечения для контактных лиц, относящихся к группе повышенного риска. Среди других ключевых направлений деятельности – внедрение системы мониторинга и оценки мероприятий по ликвидации лепры, переход медицинской информационной системы на платформу DHIS2 и повышение квалификации врачей общей практики и дерматологов для улучшения диагностики и своевременного направления к специалистам. Стратегия также подчеркивает особую важность предоставления консультационной поддержки вновь выявленным пациентам и активное участие лиц с лепрой в обсуждениях соответствующей политики. Этот план действий предлагает масштабируемые и релевантные на общемировом уровне стратегии ликвидации лепры в условиях низкой эндемичной заболеваемости. Опыт Пакистана и накопленные знания могут послужить источником информации и примером для подобных действий в других странах.

## Introduction

The chronic infectious disease leprosy, caused by *Mycobacterium leprae*, still contributes to preventable disability worldwide.^1^ *M. leprae* infects Schwann cells, causing severe axonal damage in peripheral nerves and leading to chronic peripheral neuropathy characterized by loss of sensation and muscle weakness.^2^^,^^3^ Consequently, the sensory loss and related complications can lead to tissue damage and subsequent disability, which are easily recognizable in individuals affected by leprosy.^2^^,^^3^

In the 1980s, the World Health Organization (WHO) introduced a multidrug therapy with three antibiotics: rifampicin, clofazimine and dapsone, resulting in a considerable decline in the number of registered leprosy cases; however, new cases continue to occur worldwide.^4^^,^^5^ Although the disease is effectively treated with the multidrug therapy, delays in diagnosis and subsequent inflammatory episodes mean that permanent nerve damage is common, often affecting almost half of those with the disease.^2^ Stigma and discrimination are among the most debilitating outcomes for affected individuals and their families, often leading to mental health problems and social ostracism.^6^

The very long incubation period of *M. leprae*, often 5 to 10 years, or longer, poses a major challenge to early detection and prompt treatment.^7^ Timely detection of new cases, and prevention and management of inflammation are crucial to prevent disability at the individual level and to interrupt transmission within a population.^8^^,^^9^ Case detection delay is defined as the period from the onset of the first signs and symptoms to diagnosis and treatment initiation with multidrug therapy.^10^ According to the WHO classification system, leprosy primarily presents in two forms: paucibacillary and multibacillary.^11^ Paucibacillary leprosy is characterized by up to five skin lesions, with no bacilli detected in slit skin smears and typically less nerve damage. Multibacillary leprosy is characterized by numerous skin lesions, bacilli detected in skin smears and more extensive nerve damage. A prolonged delay in diagnosis results in damage to peripheral nerves, ultimately leading to visible deformities, or grade 2 disability as per WHO classification system. Grade 2 disability is considered a proxy indicator for leprosy case detection delay, and a high proportion of individuals with grade 2 disability could indicate considerable diagnostic delay in a population.^12^^,^^13^

## Leprosy elimination framework

In 2023, WHO developed a new leprosy elimination framework to support countries and subnational areas that are working towards achieving elimination of leprosy. The framework comprises three phases followed by a non-endemic status.^14^ The phases are defined by the annual number of new cases detected among adults (15 years or older) and children younger than 15 years. An important milestone is the end of phase I, marked by the interruption of transmission, which is indicated by the absence of child cases for five consecutive years.^15^ Phase 2 spans the period from the interruption of transmission to the elimination of disease, and progression to the next phase requires no new autochthonous cases for at least three consecutive years, along with no child cases during a five-year period. Phase 3 involves post-elimination surveillance, and the phase is completed when no or only sporadic autochthonous cases are reported over 10 years. After true elimination there will be no more autochthonous child cases younger than 15 years.^15^

The framework includes two new tools for monitoring progress towards elimination. First, the Leprosy Elimination Monitoring Tool is a spreadsheet-based quantitative tool which displays the numbers of new cases of adults and children in each subnational area. The tool can then be used to populate maps illustrating case numbers by district for each year. The second tool is the Leprosy Programme and Transmission Assessment,^16^ which is used to evaluate the essential elements of the programme, to ensure that any leprosy cases (either previously diagnosed cases or sporadic new cases in older adults or migrants) can be properly diagnosed and managed. 

Here we describe the leprosy control efforts undertaken in Pakistan and the proposed actions for elimination of leprosy in the country. When writing this article, we collaborated with many stakeholders, especially staff members of the Marie Adelaide Leprosy Centre and Aid to Leprosy Patients. This article builds on an unpublished internal evaluation report of the current leprosy control and elimination activities in Pakistan done in 2022, and the concept of a zero leprosy roadmap developed by the Global Partnership for Zero Leprosy.^17^

## Leprosy control in Pakistan

Pakistan, a middle-income country with a low level of leprosy endemicity,^4^ had a total population of 248 million people in 2023. Each of the seven main administrative units (four provinces and three territories) is responsible for its own health-care services, with very little involvement from the federal government. There is no official national leprosy control programme; instead, the provincial health services employ most of the field staff involved in leprosy work, and any training provided occurs with their approval. All programme activities, including training, technical oversight and supervision, are conducted by one of two nongovernmental organizations (NGOs) specializing in leprosy control in the country. These NGOs have been supported for decades by the German Leprosy and Tuberculosis Relief Association.

The NGO Marie Adelaide Leprosy Centre, with headquarters in Karachi, is responsible for leprosy control activities throughout the country, except in the Punjab province and Hazara Division of the Khyber Pakhtunkhwa province, which are covered by Aid to Leprosy Patients. More than half of the country’s population resides in areas served by the Aid to Leprosy Patients, whose headquarters are in Rawalpindi. The two NGOs function separately, but the combined leprosy statistics are forwarded to WHO by the director of Aid to Leprosy Patients, who is the focal person for leprosy in Pakistan. Both NGOs run clinics, but in most of the country, government clinics and government professionals provide the leprosy-related services. The NGOs have a good relationship with dermatologists throughout the country, who refer many cases or suspect cases to the NGO clinics.

### Epidemiological situation

**Fig. 1:**
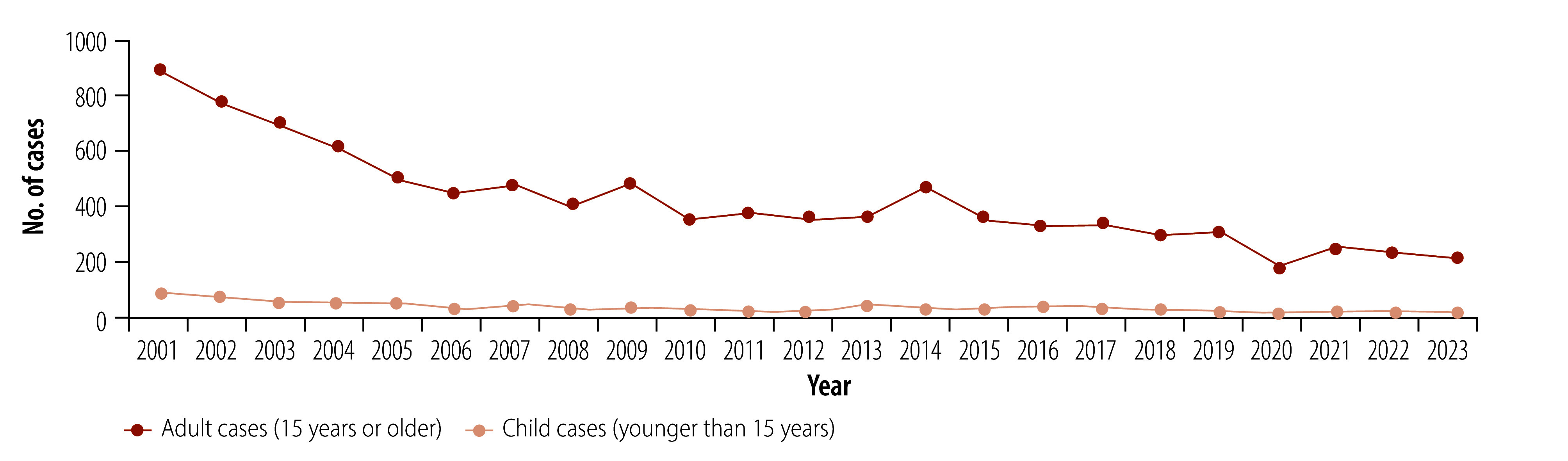
Number of diagnosed leprosy cases, Pakistan, 2001–2023

**Fig. 2:**
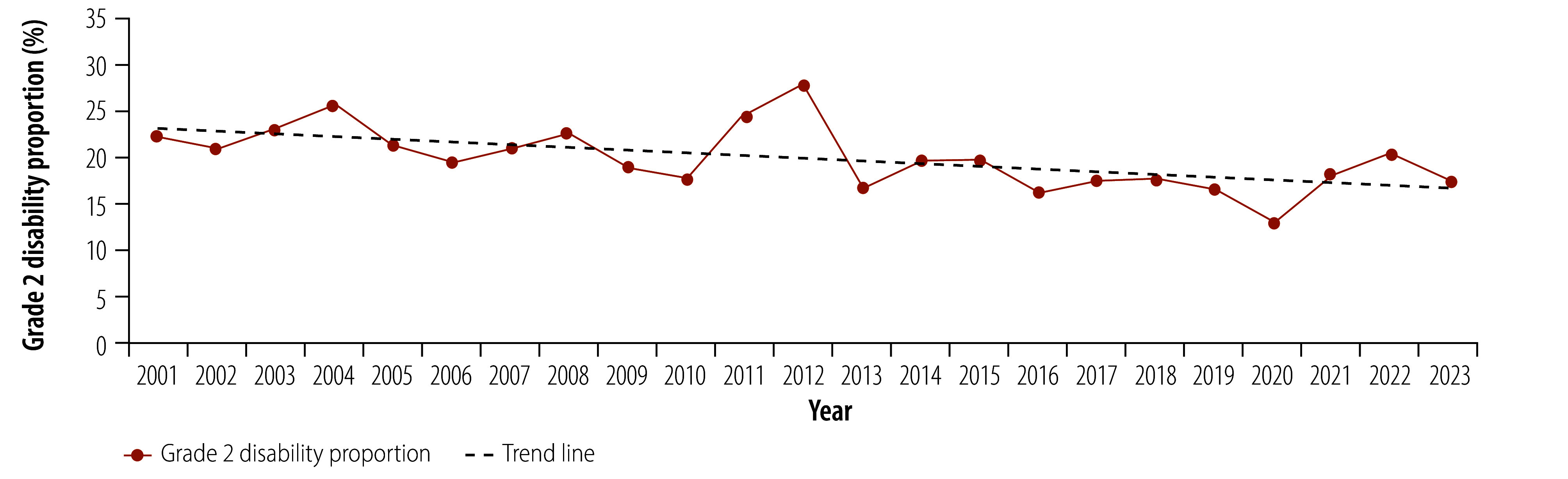
Trend in the proportion of people with grade 2 disability among new leprosy cases, Pakistan, 2001–2023

Note: we used a simple linear regression to calculate the trend line.

In 1996, Pakistan achieved the WHO target of elimination of leprosy as a public health problem (defined as a prevalence of less than 1 per 10 000 population). However, leprosy remains present in the country, with 236 new cases reported in 2023, classifying the country as having a low endemic status.^4^^,^^18^ The number of new leprosy cases has declined steadily over the past 20 years, with adult cases declining by 75%, from 878 cases in 2001 to 220 cases in 2023. The number of child cases (younger than 15 years) has declined by 83%, from 93 cases in 2001 to 16 cases in 2023 (Fig. 1). While the number of people with disability has declined substantially over this period (from 216 cases of grade 2 disability in 2001 to 50 in 2023) about one fifth of new leprosy cases present with grade 2 disability, indicating that early case detection remains a challenge (Fig. 2).

**Fig. 3:**
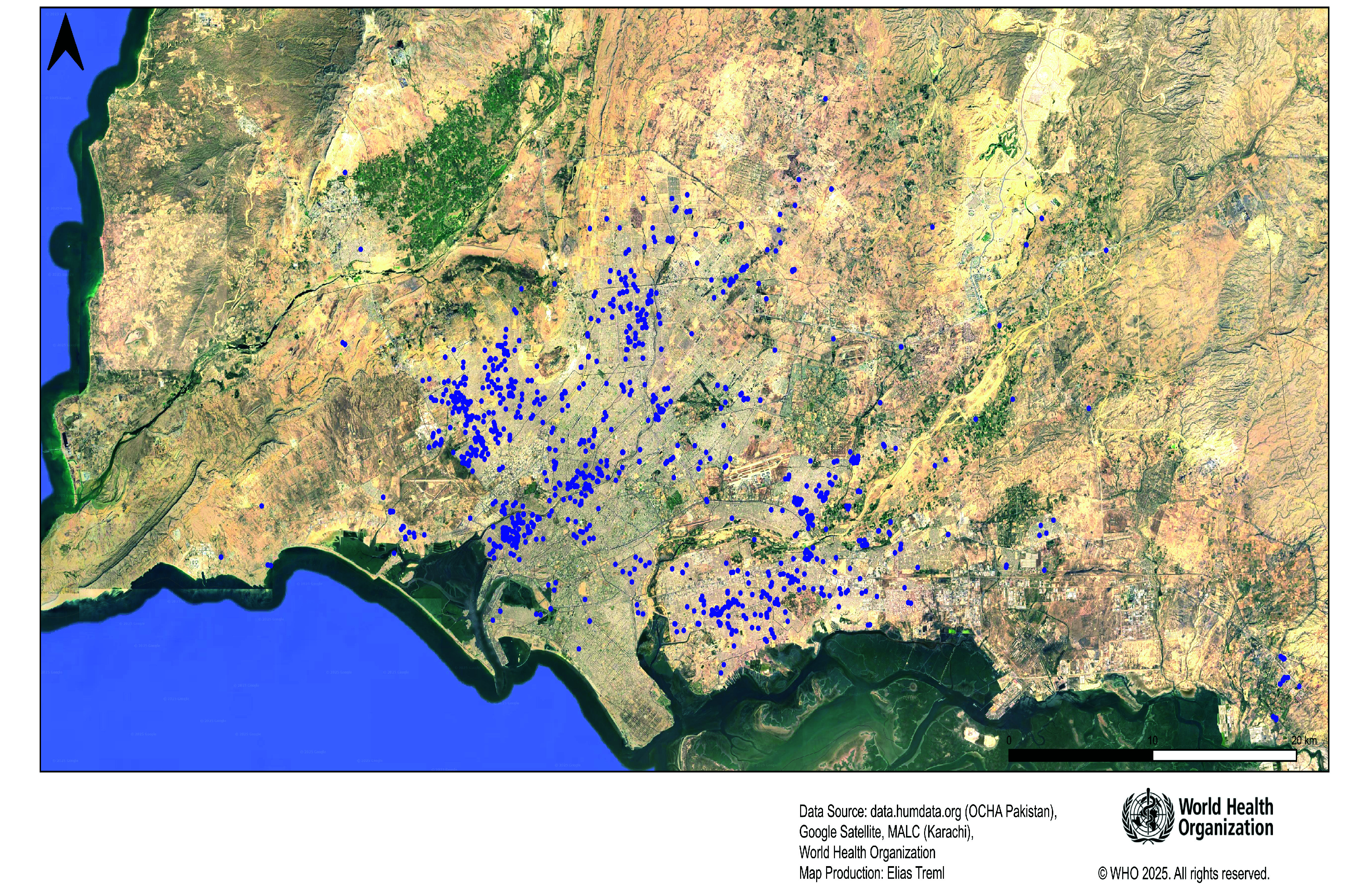
Leprosy cases detected in the Karachi area, Pakistan, 2003–2022

Note: each dot in the map represents one new case.

Using the Leprosy Elimination Monitoring Tool, we mapped leprosy cases detected from 2009 to 2022 in Pakistan (available in authors’ online repository).^19^ Karachi, the largest city in Pakistan with a population of 20 million, still has a considerable number of new leprosy cases each year, with about 50 new cases annually. Using patients’ geolocation information, we developed a map showing the distribution of new leprosy cases in Karachi from 2003 to 2022, with a random error of 100 m (Fig. 3). We also mapped the location of adult cases diagnosed between 2003 and 2008 and between 2014 to 2019, showing a concentration of newly diagnosed cases in some hotspot areas (authors’ online repository).^20^

### Leprosy elimination strategy

The main part of the zero leprosy roadmap is the leprosy elimination strategy. The key components of this strategy are outlined in Box 1. These components include increasing the efficiency of active case-finding to identify new cases as early as possible, and providing post-exposure prophylaxis to all eligible contacts after screening.^21^ To increase public awareness and cooperation, health promotion activities will be important, alongside greater involvement with those affected by leprosy in policy and practice matters. Training health workers, including general practitioners and dermatologists, will be essential to improve their capacity to accurately diagnose and refer cases.^22^ Improving data management methods will strengthen monitoring, supervision and reporting processes, and mapping leprosy cases will help target activities to hotspot areas. Additionally, integrating screening efforts with other neglected tropical diseases, particularly disabling skin conditions such as cutaneous leishmaniasis, will improve the efficiency of integrated active case-finding. Our field experiences from skin camps conducted in the Sindh and Punjab provinces in recent years have demonstrated that combining screenings for several skin conditions improves the cost–effectiveness of neglected tropical disease control activities. These integrated approaches have proven effective in identifying and managing cases more efficiently, leading to improved patient care and better resource utilization.^23^

Box 1 **Key components of the leprosy elimination strategy, Pakistan**

- Active case-finding
- Post-exposure prophylaxis for contacts
- Health promotion activities
- Engagement with those affected by leprosy
- Training additional health workers in clinical diagnosis of leprosy
- Strengthening monitoring, supervision and reporting to the German Leprosy and TB Relief Association
- Mapping of leprosy cases
- Quality assurance for diagnostic procedures
- Integration with other skin-neglected tropical diseases
- Prevention and management of disability
- Monitoring of stigma, discrimination and mental health
- Applying a human rights-based approach
- Research on local applicability and feasibility of roadmap implementation
- Physical and psychosocial rehabilitation for patients
- Sequencing and cluster analysis of leprosy cases

Note: effective treatment of leprosy with multidrug therapy (MDT) has been well established in Pakistan since the 1980s. All diagnosed cases of leprosy continue to receive effective MDT as part of the national programme.

As Pakistan moves towards elimination of leprosy, a robust quality assurance system will need to be established for laboratory-based diagnostic procedures. Since most leprosy cases in the final phases of elimination are likely to be multibacillary, collecting skin biopsies for polymerase chain reaction (PCR) analysis and, where possible, sequencing of *M. leprae* will be beneficial for transmission surveillance. With approximately 250 cases annually, performing PCR analysis and sequencing for all people with leprosy should be feasible, either in Pakistan or elsewhere. Incorporating sequencing in the national surveillance system combined with geographic mapping can help identify subnational clustering of new cases, highlighting transmission status by area.

The prevention and management of disability, along with the rehabilitation of those in need, are critical components of the strategy. Monitoring stigma, discrimination and mental health problems, and developing appropriate interventions are also priorities within the zero leprosy roadmap for Pakistan.^24^ Adopting a human rights approach ensures the dignity of all those affected by leprosy, granting them access to necessary services without discrimination. Ongoing research in Pakistan on diagnostic delays, the implementation of single-dose rifampicin post-exposure prophylaxis and transmission surveillance ensures the local applicability of interventions and supports the dissemination of generated knowledge. Rehabilitation efforts by the two NGOs in Pakistan for people affected by leprosy include both physical and psychosocial support to improve their quality of life.

The development of the zero leprosy roadmap, including the proposed leprosy elimination strategy, was a collaborative process involving local experts and international specialists with decades of experience in leprosy control. Designed to align with global best practices while addressing Pakistan’s epidemiological and health system challenges, the roadmap was developed with active engagement from local, provincial and federal government authorities. To align with existing policies and secure endorsement, we formally presented the roadmap to both provincial and national health authorities. The costing estimates for implementing the roadmap include the resources needed for capacity building; active case detection; systematic contact tracing; implementation of single-dose rifampicin post-exposure prophylaxis; patient management; and stigma reduction. While the German Leprosy and TB Relief Association will be the main funder, implementation of the roadmap will be led by Aid to Leprosy Patients and the Marie Adelaide Leprosy Centre, continuing their longstanding role in leprosy control and elimination in Pakistan. Provincial health authorities will collaborate closely through their existing health-care infrastructure to support these efforts.

## Recommendations

Our recommendations for leprosy elimination in Pakistan include improving early detection of leprosy cases by screening the contacts of confirmed patients and offering them single-dose rifampicin as post-exposure prophylaxis. This nationwide approach will be guided by geographical mapping to target areas with higher case burden. This expansion could involve innovative methods, such as skin camps and mobile health units, to deliver screening, care and prevention to remote areas.^25^^–^^27^ Using case detection delay modelling can help identify areas with substantial numbers of undiagnosed cases, enhancing the efficiency and cost–effectiveness of skin camps and other active case-finding interventions.^28^ Other approaches like a screening questionnaire with self-inspection, possibly linked to a smartphone application, can be used to find cases in very low-endemic areas. When implementing the roadmap, the NGOs should place emphasis on sustained leprosy surveillance for several years post-elimination due to the long incubation period of *M. leprae*.

While rifampicin is currently recommended for post-exposure prophylaxis, readiness to introduce more effective forms of prophylaxis such as rifapentine when they become available, is essential for the interruption of transmission and leprosy elimination.^29^ Maintaining a comprehensive database, based on data obtained from the health information management system DHIS2, to inform the Leprosy Elimination Monitoring Tool, and ensuring that remote areas with few cases are adequately monitored, will be crucial for transmission surveillance. Expanding the training of general practitioners and postgraduate dermatologists throughout the country, developing strategies to counsel newly diagnosed people and involving those affected by leprosy in policy-making and social issues are key to achieving leprosy elimination. In the coming years, separately funded research projects on leprosy transmission, antimicrobial resistance, integration with other neglected tropical skin diseases (especially cutaneous leishmaniasis) and the involvement of affected individuals in relevant issues will be explored as a part of the roadmap’s implementation. Despite significant progress towards leprosy elimination, discriminatory laws against leprosy still exist in Pakistan and should not be overlooked. Finally, treatment and rehabilitation of people affected by leprosy is important for restoring their physical abilities, dignity and social inclusion, enabling them to lead independent and productive lives. The rehabilitation offered should include vocational training, mental health services, psychosocial support, improved wound care, self-care instruction and provision of assistive devices to enhance re-integration into society.

## Challenges to implementation

While the roadmap provides a structured approach to eliminating leprosy in Pakistan, several challenges hinder its effective implementation. One key barrier is communities’ reluctance to accept the administration of post-exposure prophylaxis among household and social contacts of leprosy patients, driven by prevailing misconceptions, stigma and mistrust of the health system. Diagnostic delays and limited clinical expertise among health workers further exacerbate the issue, resulting in late-stage detection, ongoing transmission and high disability rates. Weak surveillance systems and fragmented data management, including limited integration of digital tools such as geographic information systems (GISs) and case tracking through DHIS2, impede effective monitoring and response efforts. Additionally, infrastructure limitations in remote areas make sample processing and digital interventions, such as skin apps, less feasible due to unstable cellular networks. Community distrust of international actors may also complicate interventions requiring the processing of samples abroad, such as PCR-based diagnostics. Furthermore, logistical constraints, including supply chain inefficiencies and human resource shortages, hinder the timely roll-out of interventions, while financial constraints and low political prioritization prevent the scale-up of active case-finding and preventive measures. Addressing these barriers requires a multipronged approach, including proactive community engagement, better training for health workers, strengthened data systems, adaptive implementation strategies and sustained political and financial commitment to ensure the roadmap's success.

## Proposed action plan

**Fig. 4:**
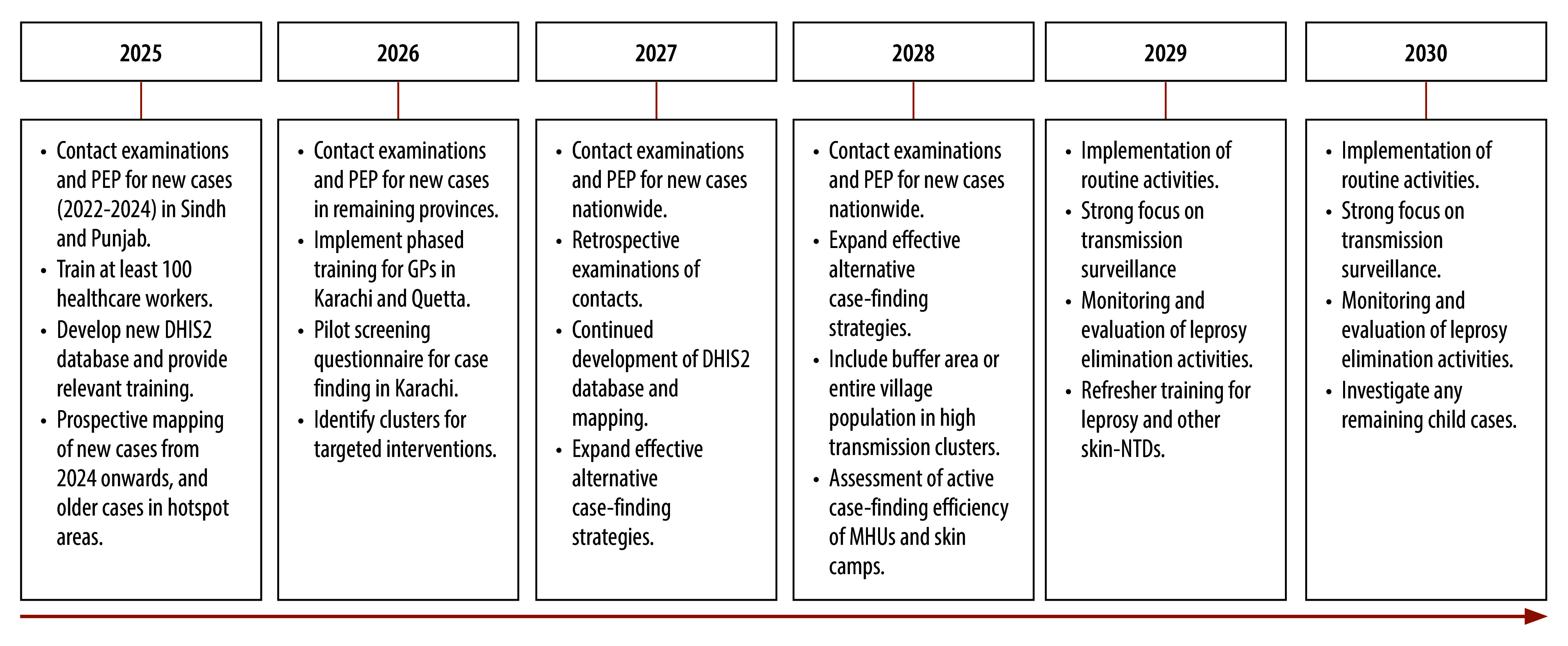
Timeline of the proposed action plan for the roadmap towards zero leprosy, Pakistan, 2025–2030

A summary of our proposed action plan for the roadmap in Pakistan is shown in Fig. 4. In 2025, the focus will be on conducting contact examinations and administering post-exposure prophylaxis to close contacts of recently diagnosed leprosy cases identified in Sindh and Punjab provinces. This approach will include close contacts in a buffer area or the entire population of villages with high transmission, using a single-dose of rifampicin or other available regimens. Additionally, Aid to Leprosy Patients and the Marie Adelaide Leprosy Centre will train at least 100 health workers to examine people and manage those with leprosy in hotspot areas, and provide remote consultations for other regions. The NGOs’ comprehensive training programmes will increase the capacity of health workers across various health-care facilities to more effectively identify and manage people with leprosy. We will develop a new DHIS2 database for the leprosy programme, along with relevant training for those collecting data in the field and those implementing the roadmap. To identify clusters for targeted interventions, such as skin camps or door-to-door, the two NGOs will geographically map new cases from 2025 onwards and older cases in hotspot areas. Another important action will be to quality assure diagnostic procedures for new cases through molecular diagnostics. 

DHIS2: DHIS2 health information system, GP: general practitioner, MHU: mobile health unit, NTD: neglected tropical disease, PEP: post-exposure prophylaxis.

In 2026, contact examinations, including contacts of newly diagnosed and known leprosy cases, and post-exposure prophylaxis administration will expand to all remaining provinces. This expansion will be facilitated by identification of case clusters for targeted interventions. We will also implement a phased training for general practitioners in Karachi and Quetta, and a pilot testing of the case-finding screening questionnaire in Karachi.

In 2027, the focus will remain on conducting contact examinations and providing post-exposure prophylaxis for contacts of all new cases nationwide, along with retrospective contact tracing and post-exposure prophylaxis for contacts of previously identified cases. Training, DHIS2 development and mapping will continue, alongside the expansion of alternative case-finding strategies including skin camps and blanket approaches, where entire communities in high-endemic, often isolated areas are screened and offered single-dose rifampicin regardless of symptoms.

In 2028, the efforts will include retrospective contact examinations and post-exposure prophylaxis administration; ensuring all contacts receive post-exposure prophylaxis; and an assessment of targeted active case-finding efforts. 

Between 2029 and 2030, we will implement routine activities, with a strong focus on transmission surveillance and the monitoring and evaluation of leprosy elimination efforts. Analysis of collected data will inform the continuous improvement of the programme.

Our observations of the development and implementation of zero leprosy roadmaps in other low-endemic countries, such as the Plurinational State of Bolivia and Togo, have highlighted key challenges and opportunities.^30^ Systematic contact examination and post-exposure prophylaxis with single-dose rifampicin are crucial for disrupting transmission, yet logistical barriers, weak surveillance and stigma continue to hinder progress. Pakistan’s roadmap serves as a model for integrating geographical mapping, molecular diagnostics and digital surveillance tools such as DHIS2. The roadmap has global relevance, offering scalable strategies for other low-endemic settings and advancing the broader goal of leprosy elimination. The lessons learnt from Pakistan’s experience provide actionable strategies that can inspire and guide similar initiatives in other countries striving to eliminate leprosy.
